# Pretreatment Neutrophil-to-Lymphocyte Ratio as a Predictive Marker of Response to Atezolizumab Plus Bevacizumab for Hepatocellular Carcinoma

**DOI:** 10.3390/curroncol28050352

**Published:** 2021-10-14

**Authors:** Yuji Eso, Haruhiko Takeda, Kojiro Taura, Atsushi Takai, Ken Takahashi, Hiroshi Seno

**Affiliations:** 1 Department of Gastroenterology and Hepatology, Graduate School of Medicine, Kyoto University, Kyoto 606-8507, Japan; htakeda@kuhp.kyoto-u.ac.jp (H.T.); atsushit@kuhp.kyoto-u.ac.jp (A.T.); takaken@kuhp.kyoto-u.ac.jp (K.T.); seno@kuhp.kyoto-u.ac.jp (H.S.); 2 Department of Surgery, Division of Hepato-Biliary-Pancreatic Surgery and Transplantation, Graduate School of Medicine, Kyoto University, Kyoto 606-8507, Japan; ktaura@kuhp.kyoto-u.ac.jp

**Keywords:** atezolizumab, bevacizumab, hepatocellular carcinoma, immune checkpoint inhibitor, vascular endothelial growth factor, neutrophil-to-lymphocyte ratio

## Abstract

Background: Combination therapy with anti-programmed death-ligand 1 monoclonal antibody atezolizumab plus anti-vascular endothelial growth factor agent bevacizumab (Atezo/Bev) was approved in 2020 as a first-line treatment for unresectable hepatocellular carcinoma (HCC). Atezo/Bev therapy is relatively well tolerated; however, factors that can predict its response have not yet been reported. Thus, we aimed to investigate whether the pretreatment neutrophil-to-lymphocyte ratio (NLR) could predict the therapeutic response in patients with HCC treated with Atezo/Bev therapy. Methods: We analyzed the course of 40 patients with HCC who received Atezo/Bev therapy at our hospital and attempted to identify pretreatment factors that could predict response by comparing those who achieved disease control with those who did not. Results: The pretreatment NLR value in patients who achieved disease control was significantly lower than that in patients with disease progression (2.47 vs. 4.48, *p* = 0.013). Using the optimal NLR cut-off value for predicting response (3.21) determined by receiver operating characteristic curve analysis, patients with NLR ≤ 3.21 had significantly better progression-free survival than those with NLR > 3.21 (*p* < 0.0001), although there were no significant differences in liver function or tumor-related background factors between the two groups. Conclusions: The pretreatment NLR value may be a useful predictor of response to Atezo/Bev therapy for HCC.

## 1. Introduction

Liver cancer is the fourth leading cause of cancer-related death worldwide, with hepatocellular carcinoma (HCC) accounting for the largest proportion [1]. HCC is still often detected at an advanced stage, and the demand for more effective and safer systemic therapies is constantly increasing. Recent advances in immunotherapy with immune checkpoint inhibitors (ICIs) have been shown to be beneficial for cancers originating from various organs [2]. In the field of HCC, combination therapy with anti-programmed death-ligand 1 monoclonal antibody atezolizumab plus anti-vascular endothelial growth factor (VEGF) agent bevacizumab (Atezo/Bev) was approved as a first line regimen for unresectable HCC in 2020 based on the randomized IMbrave150 clinical trial. The trial demonstrated that Atezo/Bev therapy is significantly superior to sorafenib with regard to progression-free survival (PFS) and overall survival (OS) [3].

Several real-world clinical studies have shown that Atezo/Bev therapy has comparable safety profiles and efficacy, as observed in the IMbrave150 study [4,5]. However, the objective response rate (ORR) and disease control rate (DCR) reported for Atezo/Bev in the updated analysis of the IMbrave150 trial are 29.8% and 73.9%, respectively [6]. Furthermore, with the availability of validated tyrosine kinase inhibitors such as lenvatinib and sorafenib as alternative first-line therapies, the identification of predictors of response to Atezo/Bev is critical to the development of appropriate treatment strategies for unresectable HCC.

The pretreatment neutrophil-to-lymphocyte ratio (NLR) has been shown to be associated with patient prognosis in a variety of carcinomas. NLR has also been reported to be associated with the response to ICIs in several carcinomas, including melanoma [7], renal cell carcinoma [8], gastric cancer [9], and lung cancer [10]. However, the importance of NLR as a predictor of the response to Atezo/Bev therapy for HCC has not yet been established. In the present study, we aimed to investigate whether pretreatment NLR could predict the therapeutic response in patients with HCC treated with Atezo/Bev therapy.

## 2. Materials and Methods

### 2.1. Study Design

All 40 patients with HCC who started Atezo/Bev therapy at the Kyoto University Hospital between October 2020 and August 2021 were included in this study. HCC diagnosis was made using dynamic contrast-enhanced CT (CECT) or contrast-enhanced magnetic resonance imaging (CEMRI). In some cases where the diagnosis could not be confirmed by imaging, pathological diagnosis by biopsy was performed. We collected patients’ baseline data, including age, sex, hepatitis virus markers, treatment history, blood cell-related markers (platelet count, NLR, platelet-to-lymphocyte ratio [PLR], and lymphocyte-to-monocyte ratio (LMR)), other serum markers (Child-Pugh grade/score, albumin, bilirubin, prothrombin, aspartate aminotransferase, alanine aminotransferase, and albumin-bilirubin (ALBI) grade/score), Barcelona Clinic Liver Cancer (BCLC) stage, and tumor markers (des-γ-carboxy prothrombin and α-fetoprotein (AFP)), before initiating treatment with Atezo/Bev. This study was approved by The Kyoto University Hospital ethics committee (R1740). The protocol of this study conformed to the guidelines of the Declaration of Helsinki. Informed consent was obtained from all patients.

### 2.2. Treatment Protocol with Atezolizumab Plus Bevacizumab

Atezo/Bev therapy (1200 mg of atezolizumab plus 15 mg/kg of body weight of bevacizumab) was administered intravenously every 3 weeks. Treatment-related adverse events (AEs) were recorded in accordance with the National Cancer Institute Common Terminology Criteria for Adverse Events (version 5.0). Atezo/Bev therapy was interrupted in patients who experienced unacceptable treatment-related AEs, according to the manufacturer’s instructions. If the only AE was grade 3 proteinuria, discontinuing bevacizumab and administering atezolizumab alone were considered acceptable. Temporary treatment interruption was maintained until the AEs resolved to a grade 1 or 2.

### 2.3. Therapeutic Efficacy

CECT or CEMRI was performed every 6–9 weeks to evaluate the therapeutic response to Atezo/Bev therapy. The category of therapeutic response (complete response (CR), partial response (PR), stable disease (SD), or progressive disease (PD) was evaluated according to the guidelines of Response Evaluation Criteria in Solid Tumors (RECIST) version 1.1. The objective response rate (ORR) was calculated as the sum of the percentage of CR and PR. The disease control rate (DCR) was calculated as the sum of the percentage of CR, PR, and SD. The PFS was calculated as the period from the start date of Atezo/Bev therapy to the date of either death from any cause or radiological tumor progression.

### 2.4. Statistical Analysis

The differences in categorical variables between the groups were analyzed using Welch’s *T*-test for continuous variables that showed normal distribution, Pearson’s chi-square test for categorical variables, or Mann–Whitney’s *U* test for continuous variables that did not show normal distribution. Receiver operating characteristic (ROC) curve analyses were performed to select the optimal cut-off value that maximized the sum of both sensitivity and specificity and calculate the area under the ROC (AUROC). Survival curves were created by the Kaplan–Meier method and compared using the log-rank test. Statistical significance was set at *p* < 0.05. Statistical analyses were performed with JMP Pro 14 (SAS Institute, Cary, NC, USA) for Windows.

## 3. Results

### 3.1. Baseline Characteristics of Enrolled Patients

The baseline clinical profiles of 40 patients (median age: 70.5 years [53–82 years], male: female = 35:5) enrolled in this study are summarized in Table 1. The number of patients diagnosed with BCLC stage B was 21 and that with stage C was 19. Thirty-four patients had received prior treatment for HCC, and six patients started Atezo/Bev as the initial therapy. A total of 26, 12, and 2 patients were diagnosed with Child-Pugh grades 5A, 6A, and 7B, respectively. Modified ALBI grade 1, 2a, and 2b were determined in 16, 12, and 12 patients, respectively. The median baseline NLR value was 2.56 (range: 0.39–14.0). The median observation period after initiation of Atezo/Bev therapy was 207.5 days (range: 29–357 days).

### 3.2. Therapeutic Response and Progression-Free Survival

**Figure 1:**
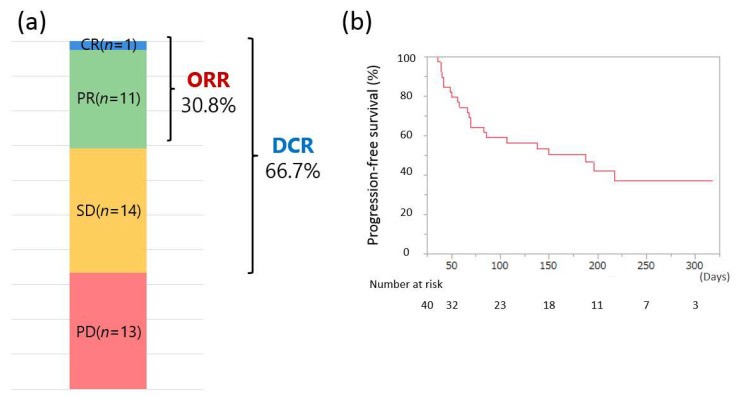
(**a**) Treatment response to atezolizumab + bevacizumab combination therapy (CR, complete response; PR, partial response; SD, stable disease; PD, progressive disease; ORR, objective response rate; DCR, disease control rate). (**b**) Progression-free survival of all 40 patients.

The therapeutic response to Atezo/Bev determined using dynamic CECT or CEMRI, according to RECIST guidelines version 1.1, is shown in Figure 1a. Of the 39 patients who were evaluated for treatment efficacy at our hospital, the ORR and DCR were 30.8% (12/39) and 66.7% (26/39), respectively, with 1, 11, 14, and 13 patients experiencing CR, PR, SD, and PD, respectively. The cumulative PFS at 50, 100, 150, and 200 days was 79.5%, 59.0%, 50.4%, and 42.3%, respectively (median PFS: 151.6 days; Figure 1b).

### 3.3. Therapeutic Response by Prior Molecular-Targeted Therapy and Etiology

**Figure 2:**
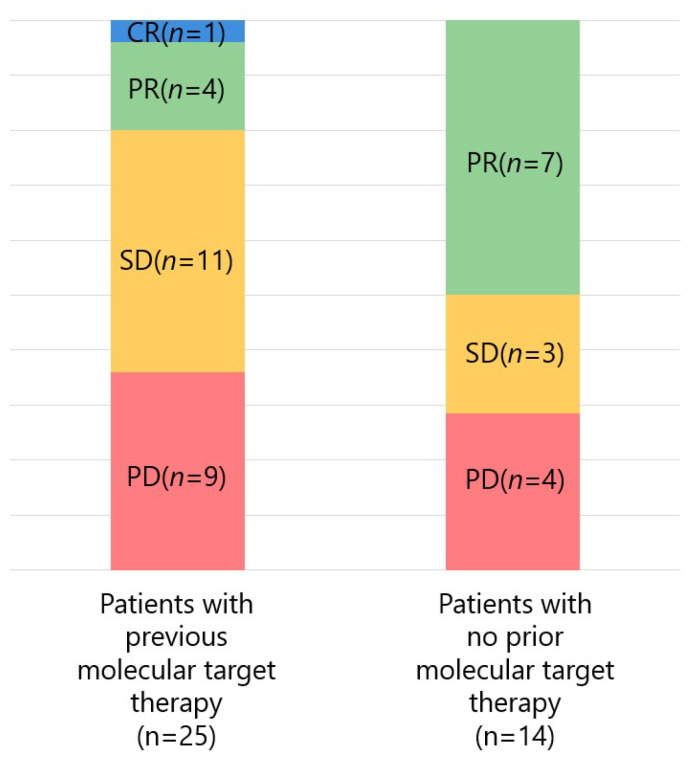
Response to atezolizumab + bevacizumab combination therapy divided by prior treatment with molecular-targeted agents (CR, complete response; PR, partial response; SD, stable disease; PD, progressive disease).

Figure 2 shows the response rate of Atezo/Bev according to the presence or absence of prior molecular-targeted therapy. Of the 25 patients with previous molecular-targeted therapy, the ORR and DCR were 20.0% (5/25) and 64.0% (16/25), respectively, with 1, 4, 11, and 9 patients experiencing CR, PR, SD, and PD, respectively. Of the 14 patients with no prior molecular-targeted therapy, the ORR and DCR were 50.0% (7/14) and 71.4% (10/14), respectively, with 0, 7, 3, and 4 patients experiencing CR, PR, SD, and PD, respectively. No significant difference was found in ORR and DCR between patients with and those without prior treatment with molecular-targeted agents (*p* = 0.052 and *p* = 0.637, respectively).

**Figure 3:**
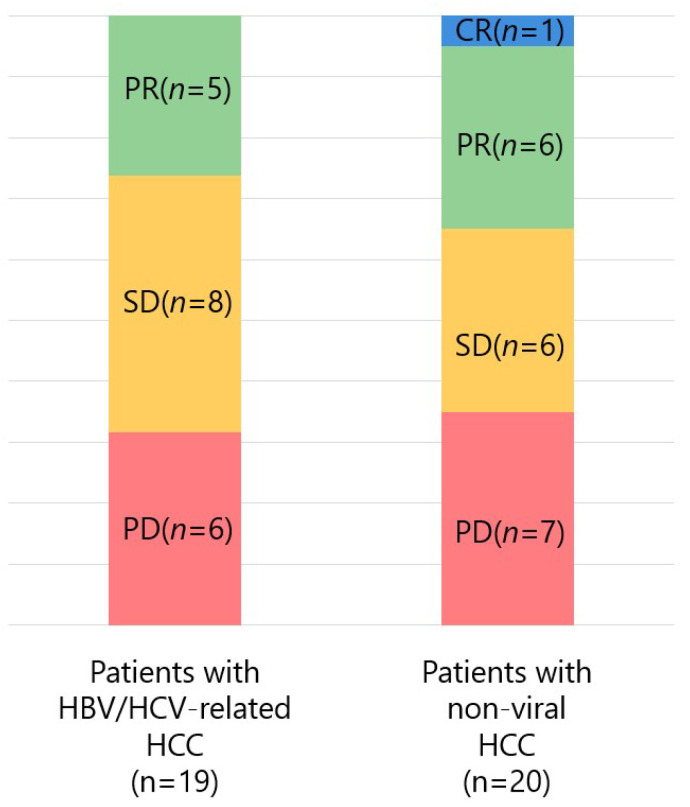
Response to atezolizumab + bevacizumab combination therapy in patients with hepatocellular carcinoma (HCC) caused by hepatitis virus infection and those with non-viral HCC (CR, complete response; PR, partial response; SD, stable disease; PD, progressive disease; HBV, hepatitis B virus; HCV, hepatitis C virus).

The response rate of Atezo/Bev by etiology was also examined (Figure 3). Of the 19 patients with hepatitis B virus (HBV) or hepatitis C virus (HCV)-related HCC, the ORR and DCR were 26.3% (5/19) and 68.4% (13/19), respectively, with 0, 5, 8, and 6 patients experiencing CR, PR, SD, and PD, respectively. Of the 20 patients with non-viral HCC, the ORR and DCR were 35.0% (7/20) and 65.0% (13/20), respectively, with 1, 6, 6, and 7 patients experiencing CR, PR, SD, and PD, respectively. No significant difference was found in ORR and DCR between patients with HCC caused by hepatitis virus infection and patients with non-viral HCC (*p* = 0.557 and *p* = 0.821, respectively).

### 3.4. Adverse Events

AEs with a frequency of 10% or more during the follow-up period with Atezo/Bev therapy are shown in Table 2. Hypertension, an AE characteristic of anti-VEGF inhibitors, was the most common (42.5%, *n* = 17; grade ≥ 3: 7.5%, *n* = 3), followed by proteinuria (40.0%, *n* = 16; grade ≥ 3: 15.0%, *n* = 6), edema (37.5%, *n* = 15), fever (32.5%, *n* = 13), fatigue (27.5%, *n* = 11), pruritus (25.0%, *n* = 10), decreased appetite (17.5%, *n* = 7), hand-foot skin reaction (12.5%, *n* = 5), rash(10.0%, *n* = 4), thyroid function abnormality (10.0%, *n* = 4), nasal bleeding (10.0%, *n* = 4), and stomatitis (10.0%, *n* = 4).

### 3.5. Comparison of Patients Who Did and Did Not Achieve Disease Control by Atezo/Bev Threapy

We compared background factors in patients who did and did not achieve disease control (CR/PR/SD vs. PD) with Atezo/Bev therapy. As shown in Table 3, the disease control group (*n* = 26) had a significantly lower AFP level before the start of treatment than the PD group (*n* = 13) (1341 vs. 7436, *p* = 0.022). In addition, the disease control group showed a significantly lower pretreatment NLR value than the PD group (2.47 vs. 4.48, *p* = 0.013).

### 3.6. Identification of NLR Cut-Off Values to Predict Disease Control

**Figure 4:**
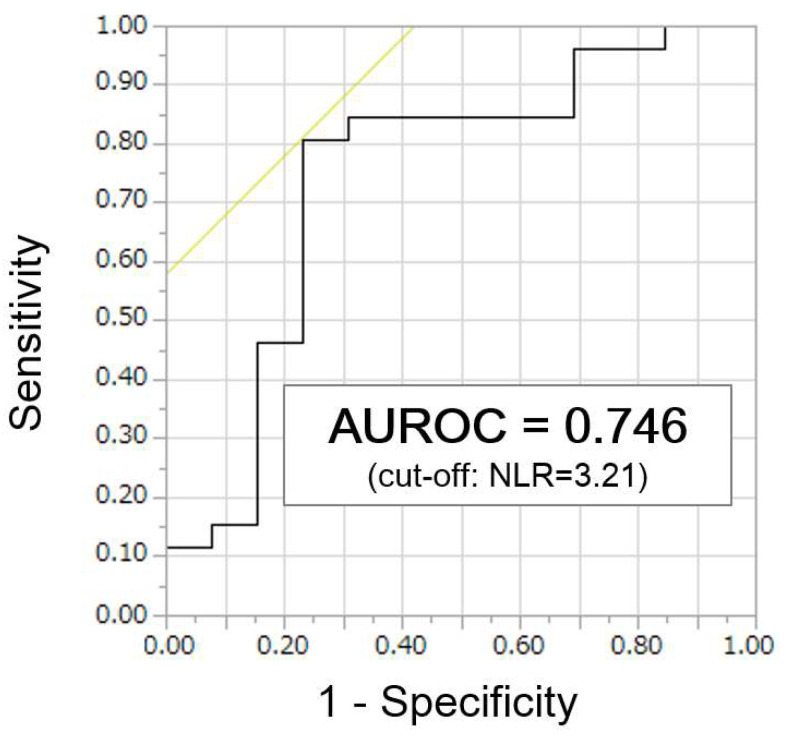
Receiver operating characteristic curve analysis to identify optimal cut-off value of neutrophil-to-lymphocyte ratio (NLR) to predict response to atezolizumab + bevacizumab combination therapy (AUROC, area under the receiver operating characteristic).

We performed ROC curve analysis on the cut-off value of NLR for predicting responses in order to further investigate the relationship between pretreatment NLR and the therapeutic efficacy of Atezo/Bev therapy. ROC curve analysis showed that the optimal cut-off value for NLR was 3.21 (sensitivity, 80.8%; specificity, 76.9%; AUROC, 0.746; Figure 4).

### 3.7. Comparison of Patients with High NLR and Low NLR

We compared background factors in patients with high NLR (*n* = 15) and low NLR (*n* = 24), according to the optimal cut-off value of NLR (3.21) determined using ROC curve analysis. The mean NLR values of the high and low NLR groups were 4.97 and 2.00, respectively (*p* < 0.0001). As shown in Table 4, there were no significant differences in liver function or tumor-related background factors between the two groups. There was a significant difference in PLR and LMR values between the two groups (*p* = 0.002 and *p* = 0.021, respectively).

### 3.8. Response Rate and Progression-Free Survival by Pretreatment NLR Values

**Figure 5:**
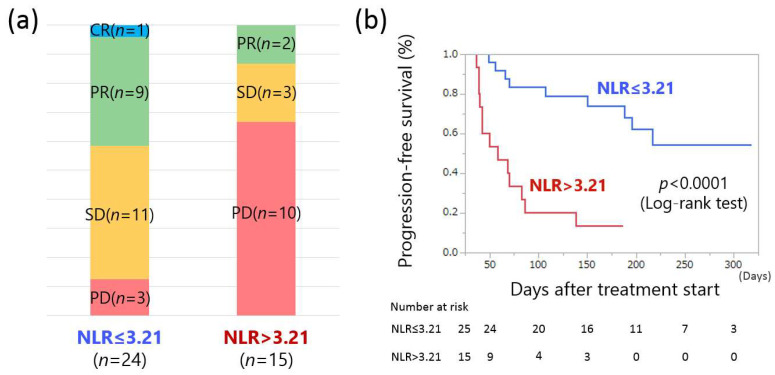
(**a**) Comparison of response to atezolizumab + bevacizumab combination therapy in pretreatment low neutrophil-to-lymphocyte ratio (NLR) and high NLR groups (CR, complete response; PR, partial response; SD, stable disease; PD, progressive disease). (**b**) Comparison of progression-free survival in the low and high NLR groups.

We further investigated the response rate and PFS of the Atezo/Bev treatment using pretreatment NLR values. Responses to Atezo/Bev therapy were 1 CR, 9 PR, 11 SD, and 3 PD in the low NLR group, and 2 PR, 3 SD, and 10 PD in the high NLR group. (Figure 5a). The ORR and DCR of the low NLR group were 41.7% (10/24) and 87.5% (21/24), respectively, while those of the high NLR group were 13.3% (2/15) and 33.3% (5/15), respectively. There was a significant difference in DCR between the two groups (*p* = 0.0005).

Cumulative PFS at 50, 100, 150, and 200 days was 96.0%, 80.0%, 64.0%, and 44.0% in patients with low NLR and 66.7%, 26.7%, 20.0%, and 0.0% in patients with high NLR, respectively. The low NLR group showed a significantly prolonged PFS compared with the high NLR group (*p* < 0.0001, log-rank test; Figure 5b). Taken together, these results suggest that the pretreatment NLR value may be a valid predictor of the response to Atezo/Bev therapy for HCC.

## 4. Discussion

To the best of our knowledge, this study is the first to show the importance of the pretreatment NLR value in predicting response to Atezo/Bev therapy for HCC. There was no difference in response to Atezo/Bev therapy according to the presence or absence of prior molecular-targeted therapy or etiology. On the other hand, the pretreatment NLR values in patients who achieved a response of SD or better on Atezo/Bev were significantly lower than those in patients who did not achieve a response (2.47 vs. 4.48, *p* = 0.013). Using the optimal NLR cut-off value for predicting response (3.21) determined by ROC curve analysis, patients with NLR ≤ 3.21 had significantly better PFS than those with NLR > 3.21 (*p* < 0.0001), although there were no significant differences in liver function or tumor-related background factors between the two groups. These results suggest that pretreatment NLR may be a valid predictor of response to Atezo/Bev therapy.

Elevated NLR has been reported to be a poor prognostic factor in a variety of solid tumors. In patients with HCC, an elevated NLR is not only a poor prognostic factor [11,12] but also a risk factor for recurrence after curative treatment including liver transplantation [13], resection [14,15], and transarterial chemoembolization [16]. NLR was devised as a marker to reflect the inflammation profile in the body in a simple way by taking neutrophil-to-lymphocyte ratio. A possible cause of the high NLR is a relatively depleted lymphocyte count in the blood and tumor. Since a low lymphocyte count reflects an impaired host immune response against tumor cells, a high NLR is speculated to be a poor prognostic factor for patients with cancer [17]. Another possible cause of elevated NLR is neutrophilia, which is associated with the high infiltration of tumor-associated macrophages and high production of inflammatory cytokines [18]. An elevated NLR in patients with HCC is associated with highly malignant tumor characteristics, such as vascular invasion, presence of multiple tumors, and high AFP levels [11]. However, notably, NLR values have been reported to be affected by various factors such as age, obesity, weight loss, steroid administration, alcohol consumption, fatty liver, malnutrition, and diabetes [12].

Pretreatment NLR has also been reported to be a predictor of the response to ICI therapy in various solid cancers. Capone et al. analyzed 97 patients with advanced malignant melanoma treated with an anti-programmed cell death protein-1 antibody nivolumab [7]. The reported that a pretreatment NLR < 4.7 was associated with significantly prolonged OS and PFS. Bagley et al. analyzed 175 patients with advanced non-small cell lung cancer treated with nivolumab and found that pretreatment NLR > 5 was significantly associated with inferior OS and PFS [10]. With regard to ICI therapy for HCC, Hung et al. reported that NLR ≤ 2.5 was a useful predictor of response to SD or better in 45 patients treated with nivolumab monotherapy, which is similar to our report [19]. On the other hand, Dharmapuri et al. reported that NLR after three cycles of nivolumab treatment was more strongly associated with the response and OS prolongation compared with NLR before treatment in 103 patients with HCC treated with nivolumab. In the present study, we did not investigate NLR fluctuation after the start of treatment, but including more cases and investigating the correlation between NLR fluctuation and treatment course are necessary.

This study has several main limitations. First, it was a single-center study with a small number of patients and short observation period. Second, all heterogeneous patients treated with Atezo/Bev at our hospital were included in this study and about half of the patients belonged to the intermediate stage. Third, the multivariate analysis was not performed due to the small sample size and the correlation between NLR and OS was not examined because of the short observation period. Therefore, the results may not be generalizable and a validation study with a larger number of patients is essential, as well as investigations of changes in NLR and continued response during Atezo/Bev therapy.

## 5. Conclusions

Pretreatment NLR may be a useful predictor of the response to Atezo/Bev therapy for unresectable HCC. Confirmation of NLR values prior to the initiation of therapy may contribute to the optimization of treatment strategies such as the selection of first line therapy and early transition to second line therapy. Further validation studies with a large number of patients are desirable. Changes in NLR values and the persistence of response after treatment initiation should be investigated.

## Figures and Tables

**Table 1:** Baseline characteristics of enrolled patients.

Characteristics	*n* = 40
Age (years, range)	70.5 (53–82)
Sex (male/female)	35/5
Etiology (HBV/HCV/non-B non-C)	6/13/21
BCLC stage (B/C)	21/19
Treatment history (naïve/recurrence) Treatment prior to Atezo/Bev Surgery TACE Lenvatinib Sorafenib Regorafenib Ramucirumab Radiation therapy	6/34 7 11 11 2 1 1 1
Aspartate aminotransferase (IU/L)	39.5 (15–192)
Alanine aminotransferase (IU/L)	28.0 (11–110)
Platelets (×10^4^/μL)	14.4 (4.2–28.1)
Child-Pugh score (5A/6A/7B)	26/12/2
ALBI score	−2.53 (−3.16–−1.45)
Modified ALBI grade (1/2a/2b)	16/12/12
α-fetoprotein (ng/mL)	19.0 (1.4–57063)
Des-γ-carboxy prothrombin (mAU/mL)	136 (12–177443)
FIB-4 index	3.57 (1.49–11.7)
Neutrophil-to-lymphocyte ratio (NLR)	2.56 (0.39–14.0)
Platelet-to-lymphocyte ratio (PLR)	125 (27.1–351)
Lymphocyte-to-monocyte ratio (LMR)	2.79 (0.86–5.41)

HBV, hepatitis B virus; HCV, hepatitis C virus; BCLC, Barcelona Clinic Liver Cancer; Atezo/Bev, atezolizumab plus bevacizumab; TACE, transarterial chemoembolization; ALBI, albumin-bilirubin; AU, arbitrary unit; FIB-4, fibrosis-4. Values are presented as median (range) or number.

**Table 2:** Adverse events during the follow-up period (>10%).

Adverse Events	Any Grade (%)	Grade ≥ 3 (%)
Hypertension	17 (42.5%)	3 (7.5%)
Proteinuria	16 (40.0%)	6 (15.0%)
Edema	15 (37.5%)	1 (2.5%)
Fever	13 (32.5%)	-
Fatigue	11 (27.5%)	-
Pruritus	10 (25.0%)	-
Decreased appetite	7 (17.5%)	1 (2.5%)
Hand-foot skin reaction	5 (12.5%)	-
Nasal bleeding	5 (12.5%)	1 (2.5%)
Rash	4 (10.0%)	-
Thyroid function abnormality	4 (10.0%)	-
Stomatitis	4 (10.0%)	-

**Table 3:** Comparison of pretreatment factors between groups of patients who achieved disease control with atezolizumab plus bevacizumab combination therapy and those who did not.

Characteristics	Patients with CR/PR/SD (*n* = 26)	Patients with PD (*n* = 13)	*p* Value
Age (years)	68.8 (7.38)	70.1 (6.46)	0.621
Sex (male/female)	23/3	11/2	0.735
Etiology (viral/non-viral)	12/14	6/7	1.000
BCLC stage (B/C)	14/12	7/6	1.000
Treatment history with MTA (yes/no)	16/10	9/4	0.637
Aspartate aminotransferase (IU/L)	53.4 (43.0)	46.7 (24.1)	0.964
Alanine aminotransferase (IU/L)	38.7 (27.0)	36.4 (20.1)	0.777
Platelets (×10^4^/μL)	16.0 (5.96)	14.2 (5.23)	0.465
Child-Pugh score (5A/6A or 7B)	18/8	7/6	0.345
ALBI score	−2.47 (0.41)	−2.32 (0.37)	0.205
Modified ALBI grade (1/2a or 2b)	12/14	3/10	0.163
α-fetoprotein (ng/mL)	1341 (5305)	7436 (15772)	0.022
Des-γ-carboxy prothrombin (mAU/mL)	12751 (40523)	2529 (3590)	0.318
FIB-4 index	4.34 (3.38)	4.83 (3.08)	0.475
Neutrophil-to-lymphocyte ratio (NLR)	2.47 (1.09)	4.48 (3.22)	0.013
Platelet-to-lymphocyte ratio (PLR)	134 (58.1)	163 (83.0)	0.270
Lymphocyte-to-monocyte ratio (LMR)	2.85 (1.08)	2.68 (1.36)	0.551

Values are presented as mean (standard deviation) or number. CR, complete response; PR, partial response; SD, stable disease; PD, progressive disease; BCLC, Barcelona Clinic Liver Cancer; MTA, molecular-targeted agent; ALBI, albumin-bilirubin; AU, arbitrary unit; FIB-4, fibrosis-4.

**Table 4:** Comparison of pretreatment factors between groups of patients with high neutrophil-to-lymphocyte ratio (>3.21) and low neutrophil-to-lymphocyte ratio (≤3.21).

Characteristics	Patients with High NLR (*n* = 15)	Patients with Low NLR (*n* = 24)	*p* Value
Age (years)	68.6 (6.54)	69.7 (7.41)	0.671
Sex (male/female)	13/2	21/3	0.940
Etiology (viral/non-viral)	8/7	11/13	0.649
BCLC stage (B/C)	8/7	13/11	0.960
Treatment history with MTA (yes/no)	10/5	14/10	0.603
Aspartate aminotransferase (IU/L)	51.5 (40.0)	51.0 (36.6)	0.762
Alanine aminotransferase (IU/L)	38.0 (24.8)	37.8 (25.1)	0.977
Platelets (×10^4^/μL)	16.4 (5.45)	14.7 (5.90)	0.411
Child-Pugh score (5A/6A or 7B)	8/7	17/7	0.268
ALBI score	−2.32 (0.40)	−2.48 (0.39)	0.298
modified ALBI grade (1/2a or 2b)	5/10	10/14	0.603
α-fetoprotein (ng/mL)	6235 (14969)	1584 (5503)	0.387
Des-γ-carboxy prothrombin (mAU/mL)	14695 (47306)	5999 (24510)	0.312
FIB-4 index	3.88 (2.39)	4.83 (3.08)	0.488
Neutrophil-to-lymphocyte ratio (NLR)	4.97 (2.69)	2.00 (0.67)	<0.0001
Platelet-to-lymphocyte ratio (PLR)	193 (77.3)	113 (38.5)	0.002
Lymphocyte-to-monocyte ratio (LMR)	2.27 (1.20)	3.13 (1.05)	0.021

Values are presented as mean (standard deviation) or number. NLR, neutrophil-to-lymphocyte ratio; BCLC, Barcelona Clinic Liver Cancer; MTA, molecular-targeted agent; ALBI, albumin-bilirubin; AU, arbitrary unit; FIB-4, fibrosis-4.

## Data Availability

The data presented in this study are available on request from the corresponding author.
